# A New Surgical Procedure “Dumbbell-Form Resection” for Selected Hilar Cholangiocarcinomas With Severe Jaundice: Comparison With Hemihepatectomy

**DOI:** 10.1097/MD.0000000000002456

**Published:** 2016-01-15

**Authors:** Shuguang Wang, Feng Tian, Xin Zhao, Dajiang Li, Yu He, Zhihua Li, Jian Chen

**Affiliations:** From the Institute of Hepatobiliary Surgery, Southwest Hospital, Third Military Medical University, Chongqing, China.

## Abstract

The aim of the study is to evaluate the therapeutic effect of a new surgical procedure, dumbbell-form resection (DFR), for hilar cholangiocarcinoma (HCCA) with severe jaundice. In DFR, liver segments I, IVb, and partial V above the right hepatic pedicle are resected.

Hemihepatectomy is recognized as the preferred procedure; however, its application is limited in HCCAs with severe jaundice.

Thirty-eight HCCA patients with severe jaundice receiving DFR and 70 receiving hemihepatectomy from January 2008 to January 2013 were included. Perioperative parameters, operation-related morbidity and mortality, and post-operative survival were analyzed.

A total of 21.1% patients (8/38) in the DFR group received percutaneous transhepatic biliary drainage (PTBD), which was significantly <81.4% (57/70) in the hemihepatectomy group. The TBIL was higher in the DFR group at operation (243.7 vs 125.6 μmol/L, respectively). The remnant liver volume was significantly higher after DFR. The operation-related morbidity was significantly lower after DFR than after hemihepatectomy (26.3% vs 48.6%, respectively). None of the patients died during the perioperative period after DFR, whereas 3 died after hemihepatectomy. There was no difference in margin status, histological grade, lymph-node involvement, and distant metastasis between the 2 groups. The 1-, 3-, and 5-year survival rates after DFR (68.4%, 32.1%, and 21.4%, respectively) showed no significant difference with those after hemihepatectomy (62.7%, 34.6%, and 23.3%, respectively). Kaplan–Meier analysis indicated that overall survival and recurrence after DFR demonstrated no significant difference compared with hemihepatectomy.

DFR appears to be feasible for selected HCCA patients with severe jaundice. However, its indications should be restricted.

## Abstract 2

Supplemental Digital Content is available in the text

## INTRODUCTION

Hilar cholangiocarcinoma (HCCA), also known as Klatskin tumor, is a type of epithelial cancer arising from the biliary confluence or right or left hepatic ducts. It accounts for more than half of cholangiocarcinomas and presents poor prognosis worldwide.^1^ The only curative treatment option is surgery. Surgical resection entails hilar bile duct resection, regional lymphadenectomy, Roux-en-Y hepaticojejunostomy, and hepatectomy. Because HCCA cells spread mainly through the bile duct, the negative bile duct margin (R0 resection) is the most important factor for radical resection. Hemihepatectomy has been widely recognized as the preferred procedure.^2,3^ Although several recent retrospective studies have reported that trisectionectomy may have a higher rate of R0 resection than hemihepatectomy,^4,5^ it needs to be validated further in additional trials. Moreover, the application of trisectionectomy is limited because of the even lower remnant liver volume (RLV).

Hemihepatectomy carries considerable operation-related risks. For the cases complicated with severe jaundice, the liver function is compromised, and the operation-related morbidity and mortality are largely amplified after hemihepatectomy. Therefore, scholars have proposed several parenchyma-preserving resection procedures, which could require radical resection, but reserve more remnant liver tissue so that the operation-related morbidity and mortality are significantly decreased.^6–9^ The major difference among different parenchyma-preserving resection procedures is the amount of the resected liver tissues. However, it has not researched agreement. Additionally, the prognosis is also under debate.

In the present study, we propose a new parenchyma-preserving resection procedure. In this procedure, liver segments I, IVb, and partial V above the right hepatic pedicle are resected together with the hilar bile duct. As the appearance of the excisional tissue is like a dumbbell, we call it dumbbell-form resection (DFR). Compared with hemihepatectomy, DFR excises sufficient bile ducts to keep the margin far enough from the tumor, but reserves more remnant liver tissues so that the operation-related morbidity and mortality might be decreased. We evaluated the data of HCCA patients who received DFR in our center from January 2008 to January 2013, and compared the perioperative parameters and prognosis with those receiving hemihepatectomy in the same period.

## PATIENTS AND METHODS

### Patients

This is a retrospective study. From January 2008 to January 2013, 184 HCCA patients received surgery at our center. Surgical procedures were choosen based on the serum examination, enhanced computed tomography, magnetic resonance cholangiopancreatography, and exploratory laparotomy. Thirty-eight patients received DFR. All of the patients receiving DFR were selected with a serum total bilirubin level (TBIL) of >200 μmol/L. In addition, the patients receiving DFR should match the above criteria: (1) the tumor should be limited in the left or right hepatic ducts, (2) without vascular invasion, (3) without liver metastasis. A total of 101patients received hemihepatectomy. Among the 101 patients receiving hemihepatectomy, 70 met the above criteria of the DFR group preoperatively and were categorized as the control group (Supplementary Figure 1). The clinical features, the information regarding preoperative percutaneous transhepatic biliary drainage (PTBD), operative parameters, and tumor pathological parameters between the 2 groups were assessed. Operation-related morbidity and mortality were analyzed. Long-term outcomes, including overall survival (from the date of surgery to last contact or death) and disease-free survival (from the date of surgery to last contact or tumor recurrence), were compared. This study was approved by the ethics committee of Southwest Hospital, affiliated to the Third Military Medical University. All patients provided informed consent.

### Preoperative PTBD

A total of 57 (81.4%) patients received PTBD in the hemihepatectomy group because of the severe jaundice (TBIL of >200 μmol/L). Severe jaundice was not the indication for PTBD in the DFR group. Although 8 patients in the DFR group did not receive immediate DFR because of obvious electrolyte disturbances or pulmonary infection, they received a period of conservative treatment before the operation. During the period of conservative treatment, these 8 patients received PTBD. Both left and right lobes were drained in patients receiving PTBD in 2 groups.

### The DFR Procedure

**FIGURE 1:**
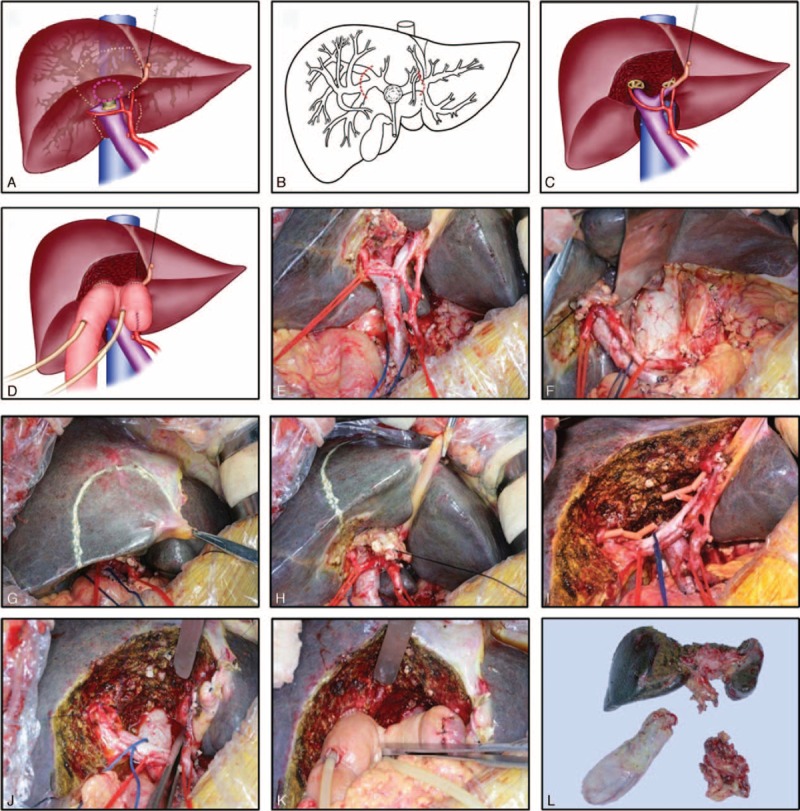
Procedure of dumbbell-form resection (DFR). Scheme of liver resection (A), proximal bile duct resection (B), proximal bile duct shaping (C), and proximal bile duct reconstructed by Roux-en-Y hepaticojejunostomy (D). (E) Hepatoduodenal ligament skeletonization. (F) Separation of caudate lobe from inferior vena cava. (G, H) Pre cut line of segments IVb and V. (I) Proximal bile duct stumps. (J) Proximal bile duct shaping. (K) Roux-en-Y hepaticojejunostomy. (L) Resected sample. DFR =  dumbbell-form resection.

Right subcostal oblique incision was used. The liver and tumor condition were evaluated by intraoperative exploration, ultrasonography, and frozen biopsy. The gallbladder was separated from the liver. Next the common bile duct was transected above the pancreas. The distal bile duct stump was closed by transfixing a suture ligature after the frozen biopsy to ensure that the stump was cancer cell negative. The fibrous, lymphatic, and nervous tissues surrounding the hepatic artery and portal vein were removed to ensure that the artery and vein were skeletonized (Figure 1E). Lymphadenectomy included the first and second station lymphnodes. When the second station lymphnodes were involved, the third station lymphnodes were also scavenged. Third station lymphnode involvement was considered to be distant metastasis. The caudate lobe was completely separated from the inferior vena cava (Figure 1F). The pre-cut line was marked (Figure 1A,G,H) and the liver tissue separation was performed by cavitron ultrasonic surgical aspirator (CUSA). Segments I, IVb, and partial V above the right hepatic pedicle were resected together with the extrahepatic bile duct. The cut line of the left bile duct was near to or exceeding the bifurcation of the third branches. The right front hepatic duct was dissected exceeding the third bifurcation. The right posterior hepatic duct was dissected near the third bifurcation (Figure 1B, I). The proximal bile duct stumps were also detected by frozen biopsy. Finally, the left or right bile duct stumps were shaped (Figure 1C,J) and were reconstructed by Roux-en-Y hepaticojejunostomy (Figure 1D,K). One to 3 latex tubes were placed exceeding the anastomotic stomas and came out from the Roux limp of the hepaticojejunostomy. As the appearance of the resected tissue was like a dumbbell, we referred to the procedure as the “dumbbell-form resection” (Figure 1L).

### Hemihepatectomy Procedures

Hemihepatectomy was performed in the same manner as DFR, except that the hepatice resection range included the left or right hepatic lobe combined with the caudate lobe. In brief, right subcostal oblique incision was used. The gallbladder was separated from the liver. Next, the common bile duct was transected above the pancreas. The distal bile duct stump was closed by transfixing a suture ligature after the frozen biopsy to ensure that the stump was cancer cell negative. The fibrous, lymphatic, and nervous tissues surrounding the hepatic artery and portal vein were removed. First and second station lymphnodes were scavenged. When the second station lymphnodes were possibly involved, the third station lymphnodes were also scavenged. The caudate lobe was completely separated from the inferior vena cava. The pre-cut line was marked and the liver tissue separation was performed by CUSA. Left lobe or right lobe was resected together with the caudate lobe and the extrahepatic bile duct. The proximal bile duct stumps were also detected by frozen biopsy. The left or right bile duct stumps were reconstructed by Roux-en-Y hepaticojejunostomy. Finally, a T type latex tube was placed exceeding the anastomotic stomas and came out from the Roux limp of the hepaticojejunostomy.

### Statistics

Data were analyzed using SPSS 17.0. Continuous data were measured by the *t* test. For categorical data, chi-squared analysis or Fisher's exact test was used. Kaplan–Meier analysis was applied for overall survival and recurrence-free survival. Statistical significance was set at a value <0.05.

## RESULTS

### Clinical Features

The clinical features of the DFR group and hemihepatectomy group are shown in Table 1. No significant differences were found between the 2 groups in terms of age and gender. The Bismuth–Corlette type distributions between the 2 groups were significantly different. The Bismuth–Corlette classification is widely used to help select the operation procedure; however, it has no prognostic value.^10^ It is noteworthy that a higher rate of patients with Bismuth–Corlette type II and IV received DFR, whereas more patients with Bismuth–Corlette type III received hemihepatectomy. Because complicated liver disease may affect the patient recovery, we next analyzed chronic HBV hepatitis, liver cirrhosis, and hepatolithiasis between the 2 groups. The number of cases complicated with liver cirrhosis in the DFR group was higher than that of the hemihepatectomy group; however, there was no significant difference. There was also no significant difference in terms of complicated chronic HBV hepatitis and hepatolithiasis.

**TABLE 1:**
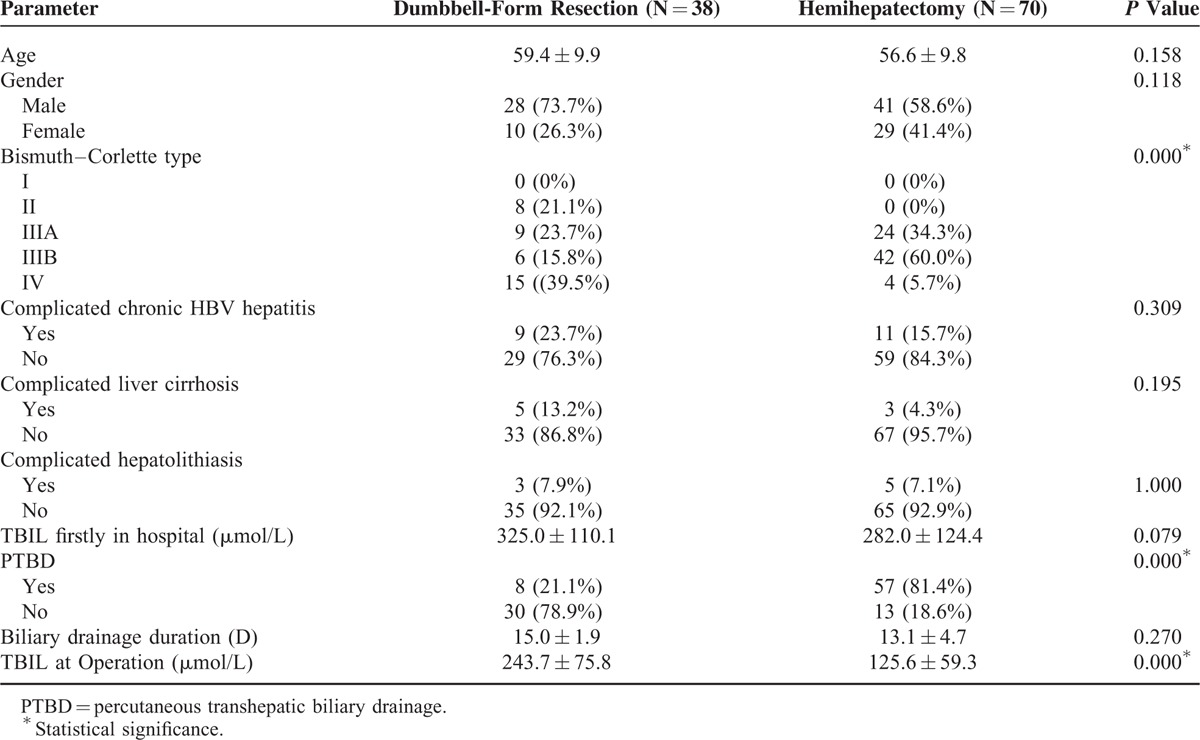
Clinical Features and Preoperative Percutaneous Transhepatic Biliary Drainage (PTBD)

### Preoperative PTBD

When HCCA patients were first in hospital, the TBIL showed no significant difference between the 2 groups (325.0 *vs.* 282.0 μmol/L, respectively. Table 1). A total of 21.1% patients (8/38) in the DFR group received PTBD, which was significantly lower than 81.4% (57/70) in the hemihepatectomy group. The drainage duration was similar between the 2 groups. Finally, the TBIL of hemihepatectomy group at operation was reduced to 125.6 μmol/L, which was significantly <243.7 μmol/L in the DFR group. The data indicated that DFR had a lower demand of PTBD for HCCA patients with severe jaundice.

### Operative Parameters

The operative parameters are shown in Table 2. There was no significant difference between the 2 groups in terms of blood loss and transfusion. Although DFR was more complex than hemihepatectomy, the operation time showed no significant difference between the 2 groups, indicating that the complexity of DFR had limited impact because of the mature operation skill. RLV is considered to be an important index for predicting the operation risk and post-operative recovery. The RLVs of the DFR group were all >70% compared with all <70% in the hemihepatectomy group (*P* < 0.001), indicating that DFR might have a low operation risk and fast post-operative recovery compared with hemihepatectomy.

**TABLE 2:**
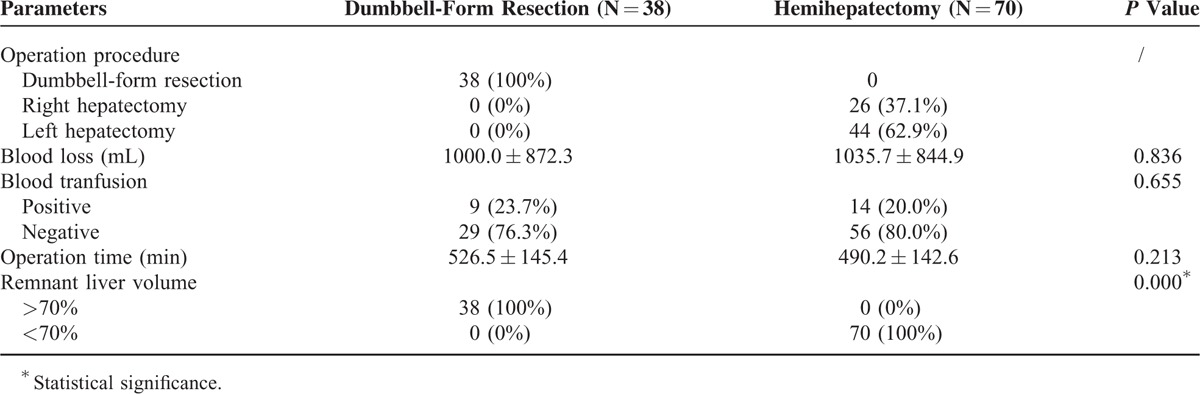
Operative Parameters

### Operation-Related Morbidity and Mortality

The operation-related morbidity after DFR was significantly lower than after hemihepatectomy (26.3% vs 48.6%, respectively; *P* = 0.025; Table 3). Notably, no patients had liver dysfunction after DFR compared with 4 patients (5.7%) after hemihepatectomy. Bile leak of the DFR group seemed to be more than that of the hemihepatectomy group (13.2% vs 11.4%, respectively), possibly because of the more proximal bile duct stumps. Three cases of operation-related death (4.3%) occurred in the hemihepatectomy group, including 2 due to liver dysfunction and 1 due to gastrointestinal hemorrhage. However no patients died because of surgery in the DFR group. The biochemical parameters were also analyzed (Supplementary Figure 2). Overall, the serum alanine aminotransferase (ALT) and aspartate aminotransferase (AST) levels after surgery were decreased quickly in the DFR group compared with those in the hemihepatectomy group. The TBIL and albumin (ALB) levels recovered quickly in patients after DFR. Consistently, the prothrombintime (PT) level recovered quickly in the DFR group. Together, the biochemical analysis data indicated that patients after DFR have a faster liver function recovery than those after hemihepatectomy. The hospital stay after DFR was significantly shorter than that after hemihepatectomy (15.2 vs 20.9 days, respectively; *P* = 0.048). Together, the data indicated that patients had a lower operation-related morbidity and mortality and more rapid recovery after DFR than after hemihepatectomy.

**TABLE 3:**
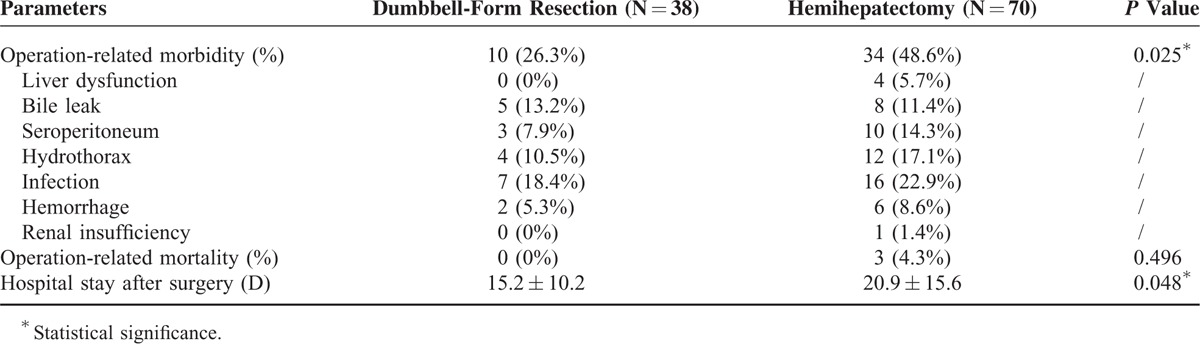
Operation-Related Morbidity and Mortality

### Tumor Pathological Parameters

The tumor pathological parameters by postoperative assessment were compared between the 2 groups. There were no significant differences in terms of histological grade, T classification, lymph node involvement and distant metastasis (Supplementary Table 1). The number of proximal bile duct stump was significantly higher in the DFR group than in the hemihepatectomy group (5.53 vs 2.13, *P* = 0.000). The negative margin rate was similar between 2 groups (76.3% vs 77.1%, respectively; *P* = 0.922). Ebata et al^11^ reported that an anatomic 10-mm margin from the tumor was required for eradication of cholangiocarcinoma. Here, we found that 29 of 38 cases (76.3%) in the DFR group showed anatomic margins >10 mm, and the rate was significantly higher than in the hemihepatectomy group (36/70, 51.4%; *P* = 0.012).

### Postoperative Overall Survival and Recurrence

**FIGURE 2:**
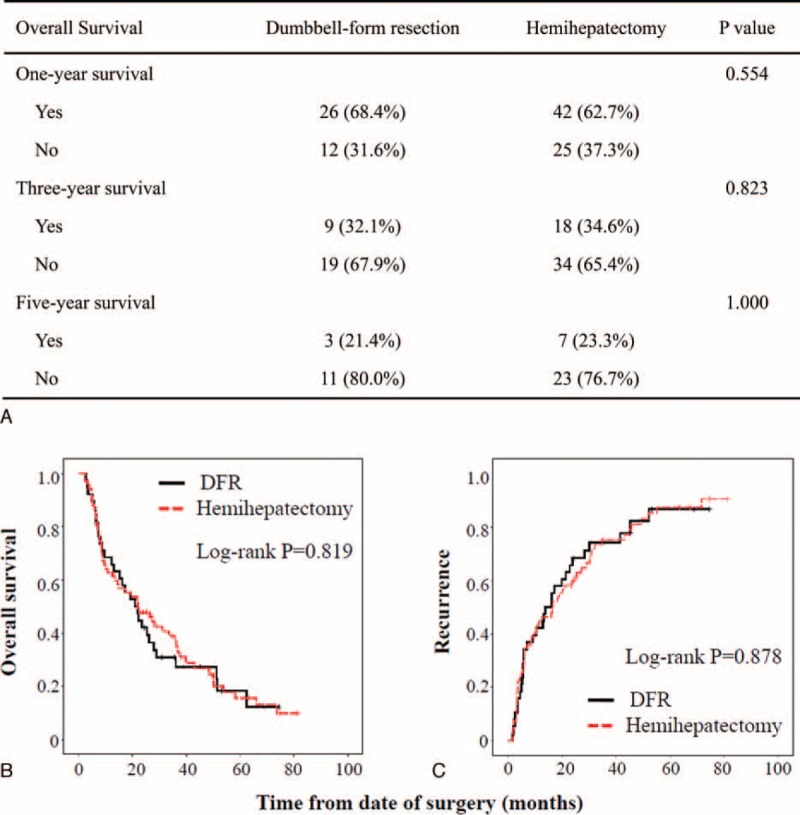
Comparison of prognosis between dumbbell-form resection (DFR) and hemihepatectomy group. (A) One-, 3-, and 5-year survival rates. Overall survival (B) and recurrence (C) analysis by the Kaplan–Meier model. DFR =  dumbbell-form resection.

Three cases in the hemihepatectomy group were excluded because of operation-related death. The 1-, 3-, and 5-year survival rates of the DFR group showed no significant difference compared with those of the hemihepatectomy group (Figure 2A, 68.4% vs 62.7%, 32.1% vs 34.6%, 21.4% vs 23.3%, respectively). Next the Kaplan–Meier model was used. The log-rank test showed that overall survival after DFR had no significant difference compared with that after hemihepatectomy (*P* = 0.819, Figure 2B). There was also no significant difference in tumor recurrence between the 2 groups (*P* = 0.878; Figure 2B). The data indicated that DFR had similar survival with hemihepatectomy for selected HCCA patients with severe jaundice.

## DISCUSSION

Hemihepatectomy has been considered the preferred curative procedure for HCCA patients. However, for most HCCA patients with obstructive jaundice, the liver function is compromised and the operation-related morbidity and mortality is further increased after hemihepatectomy.^12–14^ Clinically, preoperative biliary drainage (PBD) is widely used to decrease the TBIL level and reduce the operation risk.^15^ At our center, HCCA patients with a TBIL level above 200 μmol/L are considered to receive PTBD before major hepatectomy, and the strategy is similar with several other centers.^16,17^

However, the disadvantages of PBD should be noted. First, its impact on survival is controversial. Several studies have shown that PBD does not decrease the overall postoperative mortality in jaundiced HCCA patients.^17–19^ Second, PBD presents complications, including cholangitis, pancreatitis and even implantation metastasis.^20,21^ Third, it causes a surgery delay, the duration of which ranges from 2 to 8 weeks, and even longer in patients with liver cirrhosis, which occurs more commonly in China because of its high population of HBV infection. Thus, patients are required to have very good compliances and will suffer increased risk of losing the surgical opportunity. Fourth, PBD may necessitate additional financial and social resources, which cannot be ignored in most developing countries. Because of the lower demand of PBD, DFR could avoid most of the above disadvantages. In our experience, DFR might be safe for most HCCA patients with a TBIL <500 μmol/L without chronic basal liver diseases.

Another advantage of DFR for HCCA patients with severe jaundice is the lower operation-related morbidity and mortality. Liver failure is one of the most severe complications that may cause patient death. In the present study, DFR caused no liver failure and patient death even under the condition of higher preoperative TBIL levels. Additionally, most other complications were also fewer in the DFR group than in the hemihepatectomy group. This outcome might be due to the high RLV and fast recovery of liver function after DFR. Thus, DFR may produce a less painful recovery and have a wider adaptability for HCCA patients with severe jaundice.

Complete resection of the tumor tissue is the premise to obtain an ideal prognosis. The characteristic HCCA growth pattern mainly includes extension along the bile ducts, and the main recurrence pattern is bile duct stump recurrence.^22^ Thus, excision of the bile duct with a negative margin may result in cure.^23^ The length of the anatomic ductal-free margin is also an important factor associated with local recurrence.^4^ The aim of liver resection in DFR is to expose left and right bile ducts at a high degree, which may get high rate of R0 resection. These might be reasons for that DFR obtain a similar prognosis with hemihepatectomy in the present study. However, this finding should be validated in more clinical trials.

The indication of DFR should be restricted. In our opinion, first, the tumor should be limited in the left or right hepatic ducts. In DFR, the proximal bile duct stumps are near to or exceeding the third bifurcations, and a certain length of the anatomic tumor-free margin should be maintained. Based on this point, DFR might be suitable for most Bismuth–Corlette type II, partial Bismuth–Corlette type III and IV HCCAs. Second, patients with vascular invasion should be excluded. These patients have a high possibility of liver metastasis and major hepatectomy combined with vascular resection and reconstruction might be a better option.^24^ Third, patients with liver metastases should also be excluded. Major hepatectomy might be more appropriate. Under the above criteria, DFR may achieve an ideal prognosis. Finally, all of the above criteria can be evaluated by preoperative imaging examination combined with intraoperative exploration, ultrasonography, and frozen biopsy.

In addition to complicated severe jaundice, DFR may also be suitable for other high-risk conditions. HCCA with a small RLV is a contraindication for hemihepatectomy. Preoperative portal vein embolization (PVE) is now widely applied to increase the remnant volume and decrease the surgical risk.^25,26^ However, PVE has similar disadvantages as PBD.^27^ Immediate DFR might be another option for these high-risk HCCA patients. However, this should be investigated in more trials.

Besides DFR, some other parenchyma-preserving resection procedures have been proposed. However, the indication might be different. Noji et al^8^ reported the hilar plate resection (HPR). In this procedure, the extrahepatic bile duct is resected at the level of the hilar plate. Because HPR does not excise any liver tissue, it might be mainly applied in Bismuth–Corlette type I HCCA. Tan et al^9^ showed the combined caudate lobe and high hilar resection (CCHR), a type of parenchymal resection of approximately 0.5–1.0 cm of the hilar portion combined with the caudate lobe, for Bismuth–Corlette type IV HCCA. CCHR excises less liver tissue, whereas DFR excises more bile ducts at a higher level. Kawarada et al^6^ reported the “Taj Mahal” liver resection procedure, mainly including segments IVa and V with the caudate lobe. Actually, the concept of DFR is very close to that of the “Taj Mahal” procedure; however the liver tissue resection range is different. In “Taj Mahal” procedure, total segment V is resected, and partial segment VIII and IVa is separated. Therefore the incision resembles the contour of the Taj Mahal. In DFR, only partial segment V above the right hepatic pedicle is resected, and segment VIII and Iva is not separated. The aim of partial segment V resection in DFR is to expose enough right hepatic bile ducts. Besides, Kawarada et al does not analyze the prognosis of “Taj Mahal” resection. In our experience, resection of partial segment V above the hepatic pedicle is sufficient to excise bile ducts at a similar level compared with that of total segment V resection. Mesohepatectomy is another parenchyma-preserving resection procedure.^28^ In mesohepatectomy, segments IV, V and VIII are resected. The RLV is lower than that of DFR. Therefore, mesohepatectomy is also limited for high-risk HCCA patients. Moreover, as the total right anterior bile duct branch is excised, mesohepatectomy is particularly suitable for Bismuth–Corlette type IIIA HCCA involving the right anterior bile duct branch.

In conclusion, our data indicated that DFR appears to be safe and feasible for selected HCCA patients with severe jaundice. It provided similar long-term survival to hemihepatectomy, but had a lower demand of PBD, a faster postoperative recovery and a lower perioperative morbidity and mortality. However, its indications should be restricted for tumors limited in the left or right hepatic ducts without vascular involvement and liver metastasis.

## Supplementary Material

Supplemental Digital Content
