# Supplementary material for: A New Surgical Procedure “Dumbbell-Form Resection” for Selected Hilar Cholangiocarcinomas With Severe Jaundice: Comparison With Hemihepatectomy
Source: Medicine (Baltimore). 2016 Jan 15;95(2):e2456. doi: 10.1097/MD.0000000000002456 (PMC4718265; doi:10.1097/MD.0000000000002456)
Supplement: Supplemental Digital Content [file medi-95-e2456-s001.pdf]

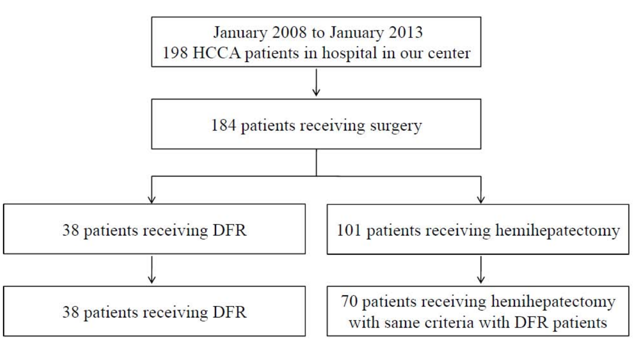
January 2008 to January 2013
198 HCCA patients in hospital in our center
184 patients receiving surgery
38 patients receiving DFR
101 patients receiving hemihepatectomy
38 patients receiving DFR
70 patients receiving hemihepatectomy
with same criteria with DFR patients

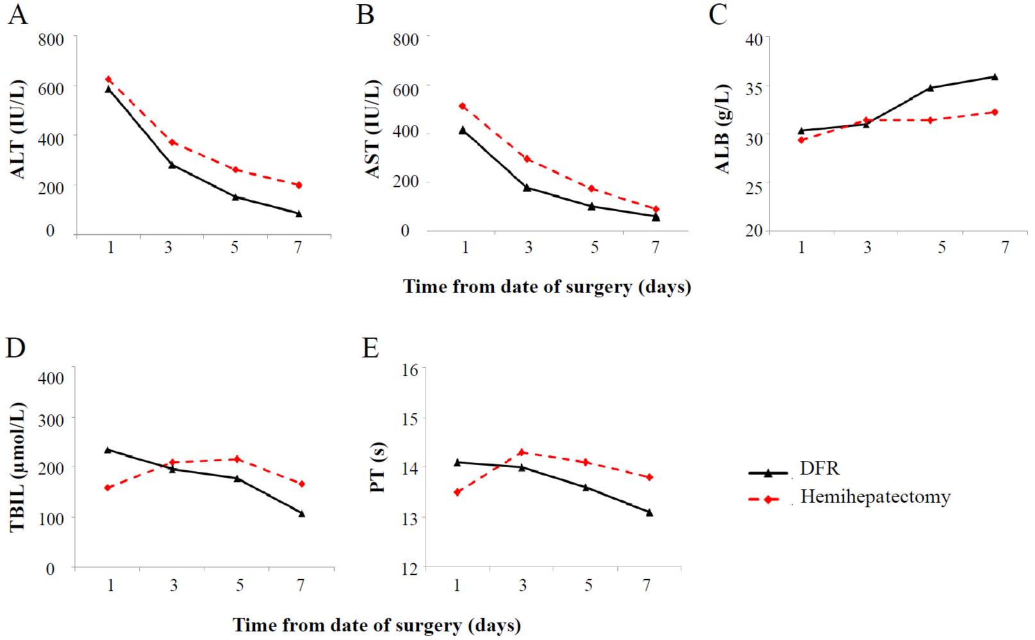

A
800
600
400
200
0
ALT (IU/L)
1
3
5
7
B
800
600
400
200
0
AST (IU/L)
C
40
35
30
25
20
ALB (g/L)
Time from date of surgery (days)
D
400
300
200
100
0
TBIL (μmol/L)
E
16
15
14
13
12
PT (s)
DFR
Hemihepatectomy
Time from date of surgery (days)

Supplementary Table 1 Tumor pathological parameters

Parameters	Dumbbell-form resection (N=38)	Hemihepatectomy (N=70)	P value
Histological grade			0.727
G1	13 (34.2%)	22 (31.4%)	
G2	15 (39.5%)	33 (47.1%)	
G3	10 (26.3%)	15 (21.4%)	
T classification			0.268
T1	3 (7.9%)	7 (10.0%)	
T2	28 (73.7%)	41 (58.6%)	
T3	7 (18.4%)	22 (31.4%)	
T4	0 (0%)	0 (0%)	
Lymph node involvement			0.869
Yes	18 (47.4%)	32 (45.7%)	
No	20 (52.6%)	38 (54.3%)	
Distant metastasis			0.361
Yes	2 (5.3%)	9 (12.9%)	
No	36 (94.7%)	61 (87.1%)	
Number of proximal bile duct stumps	5.53±0.76	2.13±0.80	0.000*
Margin status			0.922
Positive	9 (23.7%)	16 (22.9%)	
Negative	29 (76.3%)	54 (77.1%)	
Anatomic more than 10mm margins			0.012*
Yes	29 (76.3%)	36 (51.4%)	
No	9 (23.7%)	34 (48.6%)	

# According to the 6th UICC-TNM staging.

* Statistical significance.
